# Functional Trait Space Reveals Resource Use Strategies of Woody Plants in the Lijiang River Basin

**DOI:** 10.1002/ece3.72927

**Published:** 2026-01-22

**Authors:** Bing‐Juan Duan, Shi‐Guang Wei, Lin Li, Kun‐Dong Bai, Xian‐Juan Li, Yu‐Hang Yin, Xue Wang, Yan Hu

**Affiliations:** ^1^ Key Laboratory of Ecology of Rare and Endangered Species and Environmental Protection, Ministry of Education‐Guangxi Key Laboratory of Landscape Resources Conservation and Sustainable Utilization in Lijiang River Basin Guangxi Normal University Guilin China; ^2^ School of Life & Environmental Sciences Guilin University of Electronic Technology Guilin China

**Keywords:** functional trait space, life form, Lijiang River, resource utilization strategy

## Abstract

Understanding how plants allocate resources under environmental constraint is central to trait‐based ecology. In this study, we analyzed functional leaf traits of 205 woody species in the Lijiang River Basin, with a particular focus on the resource utilization strategies employed by arboreal species within this region. Furthermore, a comparison of the functional traits of plants in the Lijiang River Basin and the Yangtze River Basin has enabled the characterization of the latter's utilization of resources. The Lijiang River Basin, a karst region in southern China characterized by shallow soils and strong edaphic filtering, was measured for morphological and elemental traits across trees and shrubs and compared their functional trait spaces both within the basin and with species from the Yangtze River Basin. Both trees and shrubs in Lijiang exhibited conservative strategies, with high leaf dry matter content and low specific leaf area, reflecting adaptation to nutrient‐poor, drought‐prone soils. Despite this convergence, shrubs showed greater dispersion in trait space, suggesting more flexible responses to microhabitat variability. Shrubs occupied a larger proportion of unique trait space (18.98%), reflecting differentiation in growth form, rooting depth, and structural investment. Functionally distinct species—those furthest from the shared trait centroid—likely reflect ecological specialization within constrained environments. Comparisons with the Yangtze Basin revealed broader biogeographic divergence: Lijiang species were more acquisitive, while Yangtze species emphasized stress tolerance and resource conservation. These findings underscore how local environmental filtering and regional ecological context jointly shape plant functional strategies and highlight the role of life‐form differentiation and rare strategies in maintaining functional diversity under environmental stress.

## Graphical abstract

We found that both trees and shrubs in Lijiang exhibited conservative strategies; shrubs showed greater dispersion in trait space. Lijiang species were more acquisitive, while Yangtze species emphasized stress tolerance and resource conservation.

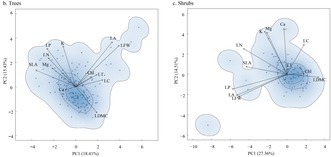

## 1 Introduction

Plant functional traits—morphological, physiological, or chemical characteristics that influence plant survival, growth, and reproduction—are widely used to understand how species adapt to environmental conditions and allocate resources (Díaz et al. 2016; Wright et al. 2004). Among these, leaf traits play a central role, as leaves are the primary site for photosynthesis, gas exchange, and nutrient cycling. Structural traits such as specific leaf area (SLA), leaf dry matter content (LDMC), and leaf thickness (LT), along with chemical traits like leaf carbon (LC), nitrogen (LN), and phosphorus (LP) concentrations, reflect key tradeoffs between rapid resource acquisition and long‐term conservation (Wright et al. 2001, 2002; Lisa et al. 2011). These leaf traits integrate plant physiological responses with environmental constraints and are especially informative in evaluating ecological strategies across environmental gradients and habitat types (McIntyre et al. 2009; Zhang et al. 2020; Michele et al. 2025).

One influential framework that organizes variation in leaf functional traits is called the leaf economic spectrum (LES), which captures a continuum from fast‐growing, resource‐acquisitive species to slow‐growing, conservative ones (Wright et al. 2004). Species with high SLA, elevated photosynthetic rates, and low tissue density typically pursue rapid returns on resource investment, while those with thicker, denser leaves and high LDMC adopt strategies geared toward persistence under stress (Zhang et al. 2023; Alamgir et al. 2022). However, ecological strategies are rarely determined by a single axis. In reality, species occupy complex positions within a multidimensional trait space, shaped by multiple interacting constraints and tradeoffs (Walker et al. 2023). Understanding how species and functional groups are distributed within this space—both within and across ecosystems—can provide deeper insights into ecological filtering, niche differentiation, and plant responses to environmental heterogeneity (Carmona and Beccari 2025).

Functional trait space provides a quantitative framework for assessing how species differ in their ecological strategies. By mapping species into a multidimensional space defined by key traits, researchers can evaluate the degree of niche overlap, functional redundancy, and phenotypic differentiation within and between communities (Hutchinson 1957; Violle et al. 2017; Mouillot et al. 2005). The size and shape of this trait space—the functional hypervolume—reflect both the diversity of strategies present and the constraints imposed by environmental filters. Comparing the functional spaces occupied by different life forms or across geographic regions helps reveal how evolutionary and ecological processes structure biodiversity (Pierre et al. 2019). Yet, for many ecosystems, it remains unclear whether plant life forms such as trees and shrubs occupy distinct regions of leaf trait space, or whether they share overlapping strategies shaped by common environmental pressures.

Lijiang River Basin, located in northeastern Guangxi, China, lies within a classic karst landscape shaped by exposed bedrock, shallow and discontinuous soils, and high surface permeability. These challenging conditions limit water retention and nutrient availability, creating strong environmental filters that influence plant survival and growth (Shen et al. 2024). As a result, the plant communities in this region exhibit distinctive structural and functional traits shaped by long‐term adaptation to drought‐prone, rocky substrates (Xiang et al. 2015; Liu et al. 2020; Liu, Huang, et al. 2021). This makes the Lijiang Basin an ideal setting for examining how different life forms—particularly trees and shrubs—have adapted their resource‐use strategies within constrained ecological space.

In this study, the funspace package was utilized to construct the functional trait space of 205 woody plant species in the Lijiang River Basin and evaluate how trees and shrubs differ in their ecological strategies and niche occupancy. Plant leaf functional traits were measured, and the extent of trait overlap and differentiation between life forms was studied, including the identification of which traits contribute most strongly to adaptive variation under karst conditions. To place these findings in a broader biogeographic context, the functional trait space of plants in the Lijiang River Basin was compared to those in the Yangtze River Basin—another major subtropical watershed that shares climatic similarities but differs in geomorphology and land‐use history (Ye et al. 2021). The Yangtze Basin spans a wider range of soil types and disturbance regimes and has served as a focal point for trait‐based ecological research in China (Liu, Ding, and Huang 2021), making it a useful reference point for understanding how regional context shapes functional diversity. The objective of this study is to compare the variation ranges of shrubs and trees in the functional trait space, assess potential differences in ecological plasticity between life forms, and establish a conceptual framework of local screening‐life form differentiation in karst habitats. Specifically, we asked three main scientific questions: (1) What are the characteristics of functional trait space for trees and shrubs in the Lijiang River Basin? (2) To what extent do these life forms share or partition ecological strategy space? (3) How does functional trait space in the Lijiang River Basin compare to that of the Yangtze River Basin? This study is pioneering in its exploration of the combination of species‐level trait space and cross‐basin comparison under karst conditions. Its findings elucidate local filtering effects and life‐form‐specific strategy shifts.

Based on the environmental characteristics of the Lijiang River Basin, and the differences in its geomorphological features and land use history compared to the Yangtze River Basin, we propose the following three assumptions: (1) Due to stronger microhabitat fragmentation and greater nutrient heterogeneity in the Lijiang River Basin, plant functional traits will exhibit a wider range of combinations, leading to increased functional dispersion. (2) Shrubs will occupy a broader and more flexible trait space than trees, reflecting their capacity to colonize marginal habitats. (3) Compared with the Yangtze River Basin, plants in the Lijiang River Basin will exhibit stronger signals of resource acquisition strategies, particularly in traits such as leaf area and specific leaf area.

## 2 Materials and Methods

### 2.1 Study Site

**FIGURE 1:**
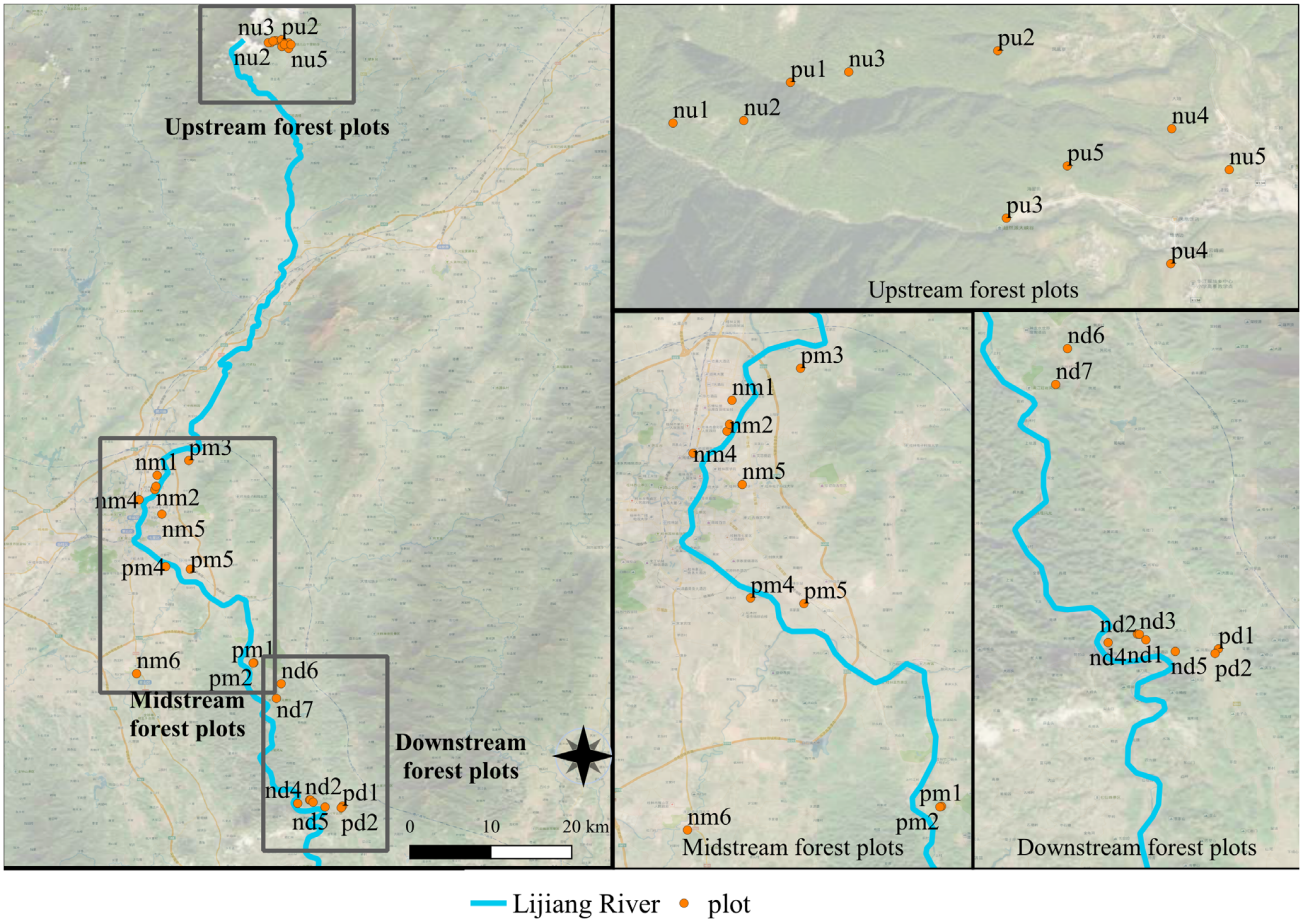
Study area (Lijiang River Basin). The names of the sample plots include letters that indicate the type and location of the forest: “n” denotes natural forest, “p” denotes planted forest, “u” denotes the upper reaches, “m” denotes the middle reaches, and “d” denotes the lower reaches.

The study was conducted in the Lijiang River Basin (24°18′–25°41′ N, 109°45′–110°40′ E), located in the northeast of Guangxi Zhuang Autonomous Region, China. The river originates in the Mao'er Mountain region of Xing'an County and extends approximately 214 km (Figure 1). The area lies within a humid subtropical monsoon climate zone, with annual precipitation ranging 1814–1941 mm. Rainfall decreases progressively from upstream to downstream. The flood season is from March to August, and the dry season is from September to February (Qin et al. 2017). The region features typical karst landforms characterized by shallow, discontinuous soils, exposed bedrock, and patchy forest distribution. Dominant soil types include red soil, with localized areas of yellow soil, paddy soil, purple soil, and bare rock (Yao et al. 2016). The Yangtze River basin is a vast geographical area, characterized by significant variations in topography and climate conditions across different regions. The present study primarily focuses on the Chongqing section of the upper Yangtze River for the purpose of comparative research. The upper reaches of the Yangtze River Basin are located within the subtropical humid monsoon region, characterized by an average annual temperature of 18.4°C, an annual precipitation of 1180 mm, and a daily maximum of 207 mm. The region experiences a prolonged period of frost‐free conditions, characterized by overcast skies and reduced sunlight. A persistent north‐easterly wind also prevails (Table 1) (Zeng et al. 2024).

**TABLE 1:** Eco‐environmental contrasts between the Lijiang and Yangtze River basins.

Environmental factors	Lijiang River	Yangtze River
Geological geomorphy	Peak clusters and depressions with shallow, discontinuous soil layers.	Low mountains, hills, and parallel ridges and valleys
Annual precipitation	1814–1941 mm, with 70%–80% concentrated between March and August, and extremely low base flows during winter	1000–1200 mm, with relatively even seasonal distribution and significant reservoir regulation
Soil nutrient	Shallow calcareous soil, high calcium, and low phosphorus	Purple soil, yellow soil, relatively rich in phosphorus and potassium
Recent land‐use history	Primarily for tourism, with limited steep‐slope farmland and preserved karst patch forests	The Three Gorges Reservoir Area has seen the expansion of afforestation programs and economic forests, with a large area of planted forests

### 2.2 Field Vegetation Survey and Data Collection

In this study, a total of 44 plots of 20 × 20 m were established in the forests of the Lijiang River Basin from 2021 to 2023. The establishment of plots and the conduct of plot surveys adhere to the guidelines established by the Center for Tropical Forest Sciences (CTFS), which has since been renamed ForestGEO. In each plot, all trees with Diameter Breast Height (DBH) > 1 cm and shrubs with height > 1.2 m were identified to species, and their DBH and height were recorded. Next, in each plot, 50–60 healthy leaves of each species were collected and brought back to the laboratory in plastic bags for subsequent determination of functional traits. In order to facilitate a comparative analysis, data collected here for the Lijiang River Basin was contrasted with that obtained from the National Ecological Data Centre for the Yangtze River Basin. This comprised data on the functional traits of 349 woody plant species (Wang, Harrison, et al. 2023).

### 2.3 Leaf Trait Measurements

Within each plot, three uniformly sized adult plants of each species were selected, and 20–30 fully expanded mature new leaves were randomly selected from each plant, and functional trait indices were measured for all species within each sample plot (three replicates for each species): Leaf area (LA), leaf chlorophyll context (Chl), leaf thickness (LT), leaf fresh weight (LFW), leaf dry weight (LDW), specific leaf area (SLA), and leaf dry matter content (LDMC). We also measured the content of several elements within leaves, including leaf carbon (LC), nitrogen (LN), phosphorus (LP), potassium (K), calcium (Ca), and magnesium (Mg) content.

To measure (LA), the leaf was scanned and measured using YMJ‐A plant leaf area measuring instrument. Leaf Chl was measured on an HM‐YB handheld chlorophyll meter. LT was measured using a vernier caliper (accuracy of 0.01 mm), and LFW and LDW were determined using a balance (accurate to 0.0001 g) to measure the weight of saturated leaves and leaves dried in a 65°C oven respectively. Specific leaf area (SLA) was calculated as SLA (cm^3^/g) = LA (cm^3^)/DW (g), while LDMC (g/g) = DW (g)/FW (g).

The content of C was determined using the potassium dichromate‐external heating method, N was measured using an automatic Kjeldahl nitrogen analyzer, and P was determined by the molybdenum antimony resistance colorimetric method after digestion with H_2_SO_4_‐H_2_O_2_. Finally, the contents of K, Ca, and Mg were determined using flame atomic absorption spectrometry after digestion with H_2_SO_4_‐H_2_O_2_ (Bao 2000).

### 2.4 Statistical Analyses

A functional trait space was drawn using the funspace package (Pavanetto and Puglielli 2024), it estimates functional space based on traits of organisms, mainly using data on the traits LA, SLA, Chl, LT, LFW, LDMC, LC, LN, LP, K, Ca, Mg. However, it should be noted that this method is not without its limitations. The study requires a substantial sample size, with a minimum of 30 species typically recommended. Smaller sample groups may be omitted during the analysis. Furthermore, it is a tool at the species level and does not support the community matrix. To know the spatial overlap of functional traits in trees and shrubs, the hypervolume (Blonder et al. 2024), geometry (Habel et al. 2024), and viridis (Simon et al. 2024) packages were used to calculate the hypervolume; they construct a hypervolume by building a Gaussian kernel density estimate on an adaptive grid of random points wrapping around the original data points. In order to more visually display the ecological strategy space of trees and shrubs, the plotly package (Sievert 2020) and the rgl package (Murdoch and Adler 2024) were used to draw the 3D map, and the cluster package (Maechler et al. 2022) was used to calculate the Gower distance.

To characterize ecological niche differences between species, the Gower distance was used. It uses trait data such as LA, SLA, Chl, LT, LFW, LDMC, LC, LP, LN. Specifically, the Gower coefficient (*G*_*jk*_) is given by:

$$G_{\mathrm{jk}}=1/n\sum_{i}^{n}\left[1-\left(\left|X_{\mathrm{ij}}-X_{\mathrm{ik}}\right|\right)/R_{i}\right]$$

where *X*_*ij*_ and *X*_*ik*_ are the numbers of individuals of group *i* in community *j* and community *k*, respectively. *R*_*i*_ is the difference between the maximum and minimum values of group *i* in all comparison communities. *n* is the total number of groups in all comparative communities. The value of *G*_*jk*_ spans from 1 (most dissimilar) to 0 (most similar).

## 3 Results

### 3.1 Functional Trait Space of Lijiang River Basin

**FIGURE 2:**
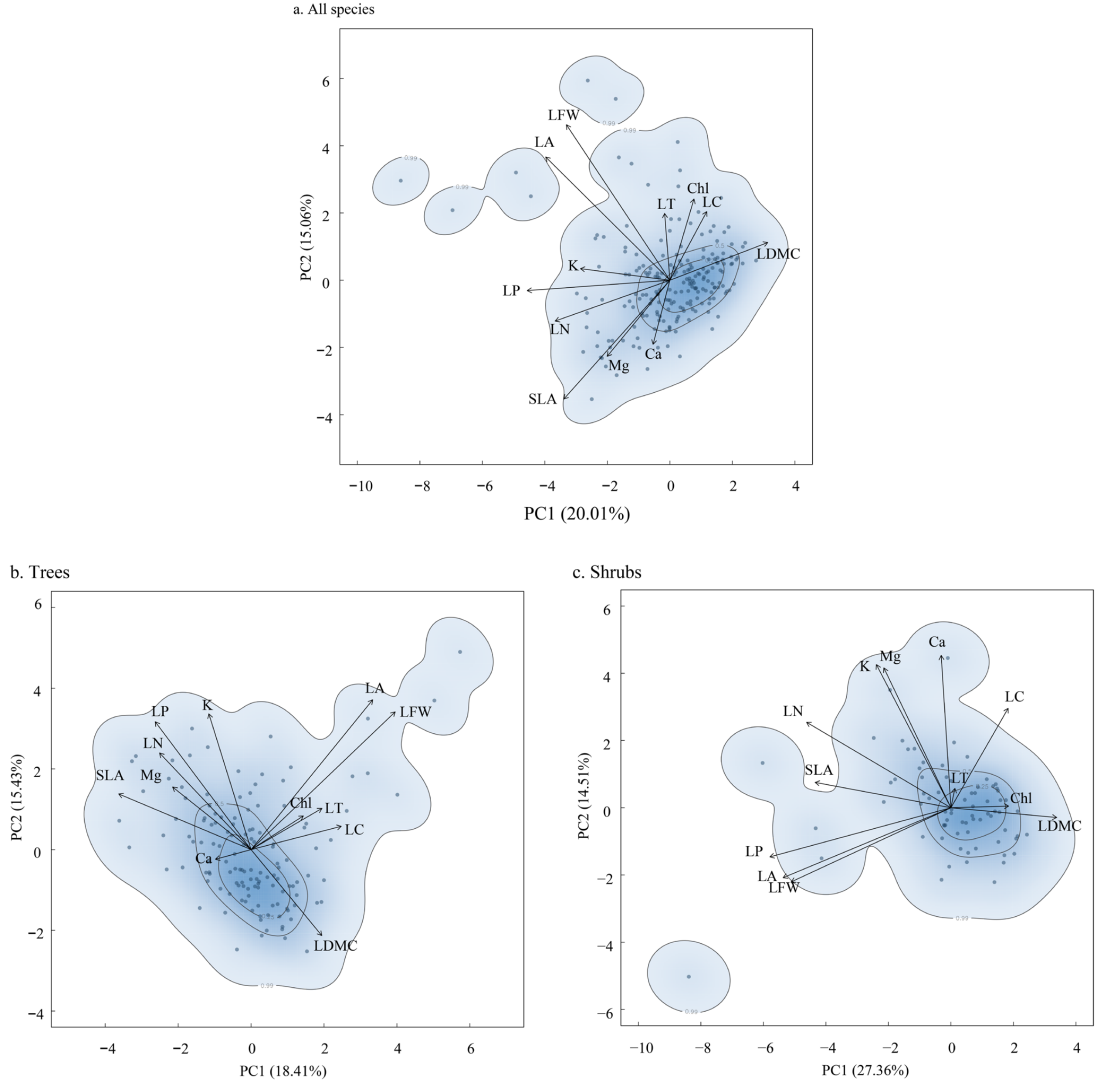
Principal component analysis (PCA) of functional trait space for woody plant species in the Lijiang River Basin. (a) All species (*n* = 205); (b) Tree species (*n* = 131); (c) Shrub species (*n* = 74). Each panel displays a kernel density estimation of species distribution in trait space. Darker colors indicate areas of higher species density. Black arrows represent the loadings and directions of functional traits on the first two PCA axes. Trait abbreviations: Ca, leaf calcium content; Chl, chlorophyll content; K, leaf potassium content; LA, leaf area; LC, leaf carbon content; LDMC, leaf dry matter content; LFW, leaf fresh weight; LN, leaf nitrogen content; LP, leaf phosphorus content; LT, leaf thickness; Mg, leaf magnesium content; SLA, specific leaf area.

For all species (*N* = 205; both trees and shrubs) in the Lijiang River Basin (Figure 2a, all woody species), the PC1 axis integrated LDMC, LA, LFW, SLA, and LP, explaining 20.01% of the variation, while the PC2 integrated LC, LN, K, Ca, and Mg and explained 15.06% of the variation. A functional hotspot was observed in the positive direction of PC1, indicating that many species have higher LDMC, Chl, and LC. Species were widely distributed along the direction of the arrow for SLA, LFW, and LA.

For trees in the Lijiang River Basin (Figure 2b), PC1 integrated LA, LFW, and Ca and explained 18.41% of the variation, while PC2 integrated Chl, LT, LN, LDMC, and SLA, explaining 15.43% of the variation. For trees, the functional trait space contains a functional hot spot distributed in the positive direction of PC2, where species tend to have higher LDMC. These tree species are widely distributed along the LFW and LA arrow direction (Figure 2b, trees).

For shrubs in the Lijiang River Basin (Figure 2c, shrubs), PC1 integrated LA, LFW, SLA, LP, and LDMC, explaining 27.36% of the variation, while PC2 integrated LC, K, Ca, and Mg, explaining 14.51% of the variation. Here, there was a functional hotspot located in the positive direction of PC1 and containing species with higher LDMC, LC, and Chl. In addition, shrub species are widely distributed along the SLA, LN, LFW, and LA arrow directions.

### 3.2 Overlapping of Ecological Strategy Spaces in the Lijiang River Basin

**FIGURE 3:**
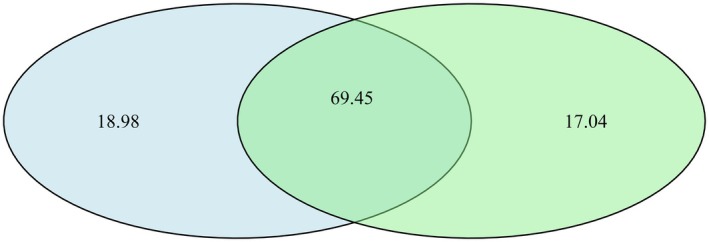
Overlap in functional strategy space uniquely occupied by tree (green) and shrubs (blue) specie. The center is where they overlap in trait space.

The area of functional strategy overlap of trees and shrubs was 69.45%, while trees occupied 17.04% of the functional strategy space alone and shrubs occupied 18.98% of the space alone (Figure 3). Several species were positioned far from the shared core of trait space, including *Dimocarpus longan* (Gower distance = 0.24), *Clerodendrum japonicum* (0.33), *Clerodendrum cyrtophyllum* (0.21), *Phyllostachys edulis* (0.21), *Callicarpa kochiana* (0.23), *Justicia adhatoda* (0.27), *Heptapleurum heptaphyllum* (0.21), *Castanopsis tibetana* (0.21), *Antiaris toxicaria* (0.21), *Eriobotrya japonica* (0.28), and *Alnus cremastogyne* (0.22), and other species are farther away from the overlapping center (Figure S1). The average Gower distance of these marginal species (mean = 0.2421) was significantly higher than that of the remaining species (mean = 0.1322; *p* < 0.001), indicating that species located at the edges of the trait space exhibited greater functional trait differentiation and reduced overlap with other species.

### 3.3 Functional Trait Space of Lijiang River Basin and Yangtze River Basin

**FIGURE 4:**
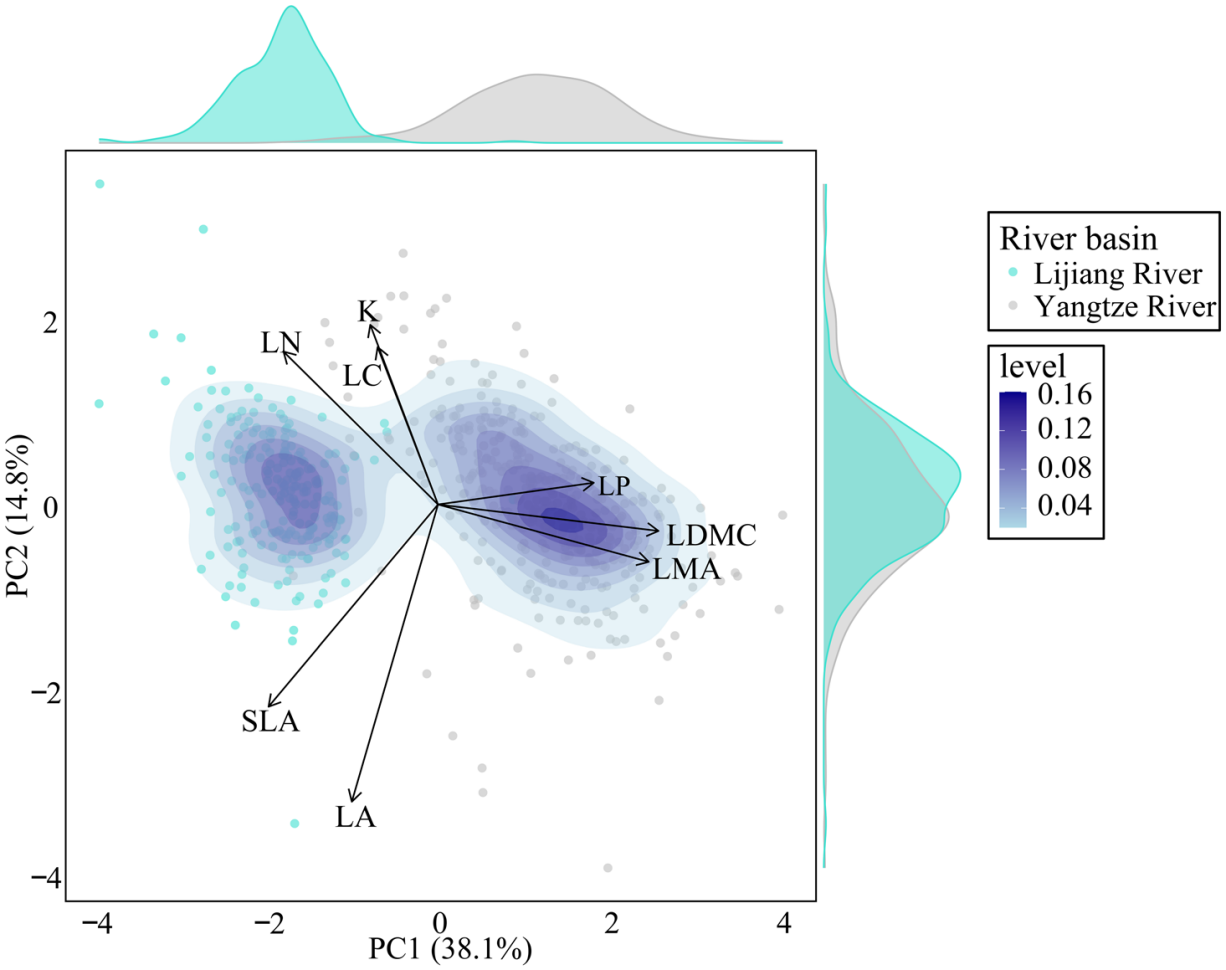
Principal component analysis (PCA) of functional trait space for woody plant species in the Lijiang River Basin and Yangtze River Basin. Displays a kernel density estimation of species distribution in trait space. Darker colors indicate areas of higher species density. Black arrows represent the loadings and directions of functional traits on the first two PCA axes. Trait abbreviations: K, leaf potassium content; LA, leaf area; LC, leaf carbon content; LDMC, leaf dry matter content; leaf thickness; LMA, leaf mass per area; LN, leaf nitrogen content; LP, leaf phosphorus content; SLA, specific leaf area.

As shown in Figure 4, the functional trait space of plants in the Lijiang River Basin and the Yangtze River Basin exhibits distinct, but partially overlapping distributions, forming a certain overlap area. The two basins share approximately 40.0% of trait space, indicating moderate similarity in overall trait composition. However, clear differences in trait dominance are apparent. Species from the Lijiang River Basin are concentrated toward the negative direction of PC1 and the positive direction of PC2, associated with high SLA and LC. In contrast, species from the Yangtze River Basin cluster toward the positive end of PC1, reflecting in higher values of LDMC and LMA. The marginal density plots further illustrate this divergence, with Lijiang species showing greater variation along PC2 and a more left‐skewed distribution along PC1 compared to the Yangtze assemblage. These patterns suggest that Lijiang species tend toward more acquisitive strategies, while Yangtze species are more conservative in resource use.

## 4 Discussion

### 4.1 Functional Strategies and Life Form Differentiation Under Environmental Filtering

The karst environment of the Lijiang River Basin imposes strong constraints on plant functional strategies, favoring traits associated with structural investment, slow turnover, and resistance to environmental stress. Traits such as high leaf dry matter content (LDMC) and low specific leaf area (SLA) were prevalent across life forms, consistent with conservative resource‐use strategies in shallow, nutrient‐poor soils (Vile et al. 2006; Liu, Huang, et al. 2021; Hou et al. 2022; Kim et al. 2025). This convergence suggests that environmental filtering limits the range of viable trait combinations across growth forms (Díaz et al. 2016; Noualhaguet et al. 2025). This has also been verified by studies in the karst region of southwestern China, where forests exhibit low specific leaf area and high dry matter content. They also reduce their investment in nitrogen and phosphorus in leaves and adopt conservative survival strategies in order to adapt to harsh habitats (Liu et al. 2022). Despite the global patchy distribution of karst landscapes, the phenomenon that the plants in the Lijiang River Basin show conservative resource utilization strategies is not widespread. Research on mixed forests in subtropical karst ecosystems indicates that strategy trade‐offs arising from leaf‐trait differentiation linked to leaf habits promote rapid resource acquisition in deciduous subcommunities and favor resource conservation in evergreen subcommunities (Wang, He, et al. 2023). The influence of environment on plant functional strategies is influenced by intraspecific variation and phylogenetic development of leaf traits. Research has indicated that incorporating intraspecific variation and phylogenetic factors serves to attenuate the apparent intensity of the environment on plant functional strategies (Siefert et al. 2015). Consequently, the present study posits that estimates of environmental filtering intensity may be overestimated due to the failure to account for intraspecific variation and the lack of phylogenetic control.

Nevertheless, shrubs and trees respond differently within this shared constraint space. Shrubs exhibited broader trait variation, especially along axes linked to SLA, leaf phosphorus content (LP), and leaf fresh weight (LFW), which relate to light interception, nutrient acquisition, and photosynthetic capacity (Kleiman and Aarssen 2007; Vance et al. 2003). This broader variation may reflect higher ecological plasticity and greater sensitivity to microhabitat heterogeneity, as shrubs often colonize exposed or fragmented substrates such as rock crevices and disturbed edges (Pfennigwerth et al. 2017; Liu et al. 2009). In contrast, trees tended to cluster around structural traits like leaf area (LA) and LFW, consistent with long‐term investment in biomass, deeper rooting, and buffered access to water and nutrients (Grime 2001; Falster et al. 2018).

Species occupying the periphery of functional space—those with combinations that diverge from both group means—may represent niche specialists. These functionally rare species can contribute disproportionately to trait space diversity and may reflect alternative strategies for coping with drought, nutrient pulses, or disturbance (Violle et al. 2017; Podani and Schmera 2006). The presence of these species in a strongly filtered system emphasizes the significance of micro‐scale differentiation and life‐history diversity in maintaining functional resilience. The importance of these species in terms of their functionality has been demonstrated in studies of shrub species in California (Ackerly 2004).

The findings of this study lend support to assumption (2), suggesting that shrubs do in fact demonstrate a greater degree of variation in functional trait space, a phenomenon that is consistent with their ecological function as colonizers of heterogeneous microhabitats. The increased aggregation of tree traits further corroborates the diversification of life forms within the ecological strategy space, constrained by shared environmental factors. In the context of forest management, it is recommended that the trait breadth maintained by shrubs—such as by conserving rock crevices and forest edge microhabitats—be utilized as an effective strategy for sustaining plant diversity in karst regions.

### 4.2 Local Divergence and Regional Context in Trait Space Structure

While the convergence of life forms within Lijiang reflects strong local filtering, comparison with the Yangtze River Basin reveals broader biogeographic divergence. Despite similar climatic conditions and partial floristic overlap, dominant strategies in the two regions diverge: Lijiang plants show a stronger signal of resource acquisition—emphasizing traits like high SLA and LA—while Yangtze Basin species are more associated with high LDMC and leaf mass per area (LMA), traits linked to long tissue lifespan and nutrient conservation (Wright et al. 2002, 2004; Liu, Huang, et al. 2021).

These differences likely reflect contrasting environmental and land‐use histories. The Yangtze region has experienced widespread agricultural development, hydrological modification, and soil degradation, leading to long‐term selection for conservative strategies suited to persistent stress (Wang 2021). In contrast, Lijiang's karst topography creates spatially fragmented habitats and ephemeral nutrient availability, selecting for a mosaic of acquisitive and conservative strategies depending on microhabitat (Liu et al. 2009, 2020).

The coexistence of convergence and divergence across scales highlights a dual structure in trait‐based assembly. Local filtering generates functional similarity among coexisting species, particularly under edaphic constraint, while regional variation in geology, disturbance, and habitat structure drives shifts in dominant ecological strategies. Recognizing both processes is essential for understanding how functional diversity is structured, maintained, and reshaped across spatial gradients.

The divergence in dominant strategies between the Lijiang and Yangtze River basins—particularly the stronger resource‐acquisition trait signals exhibited by Lijiang plants—aligns with assumption (3). Concurrently, the Lijiang River basin evinces augmented functional diversity, propelled by microhabitat heterogeneity, thereby substantiating assumption (1). However, further validation is required by integrating intraspecific variation and phylogenetic structure. Consequently, future afforestation efforts should prioritize native shrubs or early‐successional trees exhibiting high SLA and LA as acquisition‐type traits, thereby maintaining the observed resource acquisition–conservation strategy balance within the watershed.

## 5 Conclusion

This study reveals how leaf functional traits of woody plants in the Lijiang River Basin adapt to the constraints of a karst environment through conservative trait strategies. Both trees and shrubs exhibited high leaf dry matter content and low specific leaf area, reflecting convergence under strong environmental filtering. However, trees showed greater trait dispersion, suggesting more flexible responses to microhabitat variation. While trees and shrubs shared much of their ecological strategy space, they also maintained distinct functional niches—particularly through differences in traits related to structural investment and nutrient use. Functionally marginal species further expanded the community's trait diversity, potentially enhancing resilience.

Comparative analysis with the Yangtze River Basin highlighted broader regional divergence. Lijiang plants favored more acquisitive traits, likely reflecting adaptation to spatially and temporally variable soil conditions, while Yangtze species emphasized stress‐tolerant, conservative strategies. Together, these patterns underscore how both local habitat filtering and regional environmental history shape the structure of plant functional diversity.

The structure of regional scale datasets limits decomposition of intraspecific variation and phylogenetic background of leaf traits in this study. Subsequent studies should quantify the variance of intraspecific traits through individual‐scale sampling and remove the potential impact of intraspecific variation and phylogenetic signals on environmental screening by coupling phylogenetic independent comparison, so as to more accurately evaluate the intensity of environmental screening signals in karst areas.

## Author Contributions

**Bing‐Juan Duan:** conceptualization (equal), data curation (equal), validation (equal), visualization (equal), writing – original draft (equal), writing – review and editing (equal). **Shi‐Guang Wei:** conceptualization (equal), funding acquisition (equal), supervision (equal), writing – review and editing (equal). **Lin Li:** conceptualization (equal), supervision (equal), writing – review and editing (equal). **Kun‐Dong Bai:** writing – review and editing (equal). **Xian‐Juan Li:** investigation (equal), writing – review and editing (equal). **Yu‐Hang Yin:** investigation (equal), writing – review and editing (equal). **Xue Wang:** investigation (equal), writing – review and editing (equal). **Yan Hu:** investigation (equal), writing – review and editing (equal).

## Conflicts of Interest

The authors declare no conflicts of interest.

## Supporting information


**Figure S1:** ece372927‐sup‐0001‐FigureS1.png.

## Data Availability

The data used in this study is located in the figshare data repository (https://doi.org/10.6084/m9.figshare.29561486.v1) and is publicly available. The Yangtze River Basin dataset is provided by the National Ecosystem Science Data Center, National Science & Technology Infrastructure of China (http://www.nesdc.org.cn).
