# Supplementary figures and images for: Functional Trait Space Reveals Resource Use Strategies of Woody Plants in the Lijiang River Basin
Source: Ecol Evol. 2026 Jan 22;16(1):e72927. doi: 10.1002/ece3.72927 (PMC12827060; doi:10.1002/ece3.72927)

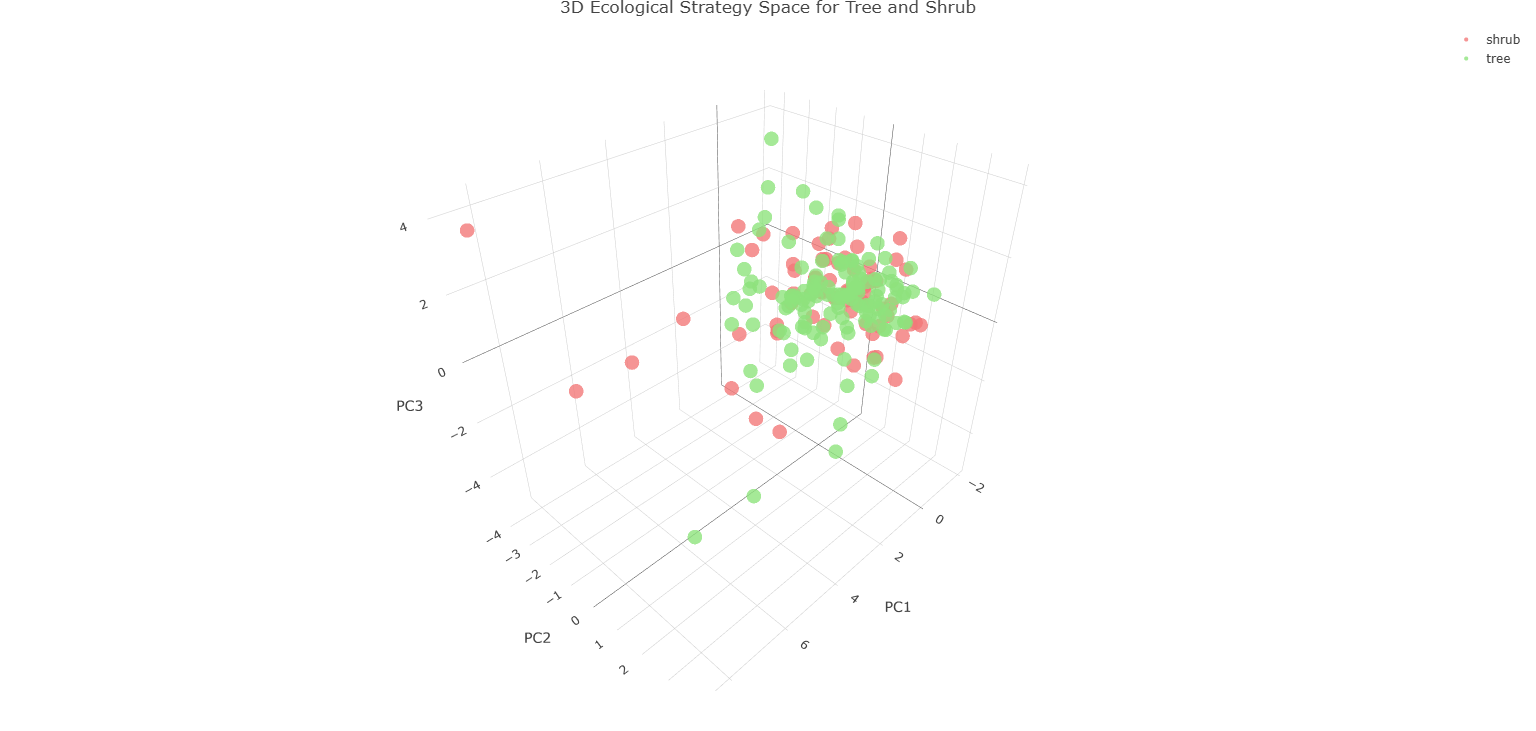

Supplement: Supplementary file 1 — Figure S1: ece372927‐sup‐0001‐FigureS1.png. [file ECE3-16-e72927-s001.png]
